# Early brain connectivity alterations and cognitive impairment in a rat model of Alzheimer’s disease

**DOI:** 10.1186/s13195-018-0346-2

**Published:** 2018-02-07

**Authors:** Emma Muñoz-Moreno, Raúl Tudela, Xavier López-Gil, Guadalupe Soria

**Affiliations:** 1 Experimental 7T MRI Unit, Institut d’Investigacions Biòmediques August Pi i Sunyer (IDIBAPS), Barcelona, Spain; 2 Consorcio Centro de Investigación Biomédica en Red (CIBER) de Bioingeniería, Biomateriales y Nanomedicina (CIBER-BBN), Group of Biomedical Imaging, University of Barcelona, Barcelona, Spain

**Keywords:** Alzheimer’s disease, Connectomics, Animal model, Magnetic resonance imaging, Resting state, Early biomarker, Transgenic, Rats

## Abstract

**Background:**

Animal models of Alzheimer’s disease (AD) are essential to understanding the disease progression and to development of early biomarkers. Because AD has been described as a disconnection syndrome, magnetic resonance imaging (MRI)-based connectomics provides a highly translational approach to characterizing the disruption in connectivity associated with the disease. In this study, a transgenic rat model of AD (TgF344-AD) was analyzed to describe both cognitive performance and brain connectivity at an early stage (5 months of age) before a significant concentration of β-amyloid plaques is present.

**Methods:**

Cognitive abilities were assessed by a delayed nonmatch-to-sample (DNMS) task preceded by a training phase where the animals learned the task. The number of training sessions required to achieve a learning criterion was recorded and evaluated. After DNMS, MRI acquisition was performed, including diffusion-weighted MRI and resting-state functional MRI, which were processed to obtain the structural and functional connectomes, respectively. Global and regional graph metrics were computed to evaluate network organization in both transgenic and control rats.

**Results:**

The results pointed to a delay in learning the working memory-related task in the AD rats, which also completed a lower number of trials in the DNMS task. Regarding connectivity properties, less efficient organization of the structural brain networks of the transgenic rats with respect to controls was observed. Specific regional differences in connectivity were identified in both structural and functional networks. In addition, a strong correlation was observed between cognitive performance and brain networks, including whole-brain structural connectivity as well as functional and structural network metrics of regions related to memory and reward processes.

**Conclusions:**

In this study, connectivity and neurocognitive impairments were identified in TgF344-AD rats at a very early stage of the disease when most of the pathological hallmarks have not yet been detected. Structural and functional network metrics of regions related to reward, memory, and sensory performance were strongly correlated with the cognitive outcome. The use of animal models is essential for the early identification of these alterations and can contribute to the development of early biomarkers of the disease based on MRI connectomics.

**Electronic supplementary material:**

The online version of this article (10.1186/s13195-018-0346-2) contains supplementary material, which is available to authorized users.

## Background

Alzheimer’s disease (AD) is a progressive age-related neurodegenerative disease that has become the most common form of dementia in elderly populations. In research for effective treatments to pause or slow the disease progression, early diagnosis is essential because currently, at the diagnosis stage, the brain has already suffered extensive damage, including accumulation of amyloid plaques, neurofibrillary tangles, and neural loss. Recent work has evidenced brain changes associated with AD starting decades prior to its clinical diagnosis [1–3]. However, the study of such preclinical stages in humans is challenging owing to the difficulties in selecting the appropriate cohorts. The late appearance of symptoms makes it difficult to identify subjects in early stages of the disease. In this regard, transgenic animal models of AD can contribute to a great degree to the identification of early biomarkers of the disease [4–7]. Despite other transgenic models of AD, TgF344-AD rats manifest all the pathological hallmarks of AD in a progressive way, including amyloid plaques, tau pathology, oligomeric β-amyloid (Aβ), neuronal loss, and behavioral impairment [8]. Other reasons to use rats instead of murine models are based on the fact that rats are physiologically, genetically, and morphologically closer to humans than mice, and their bigger brain size facilitates neuroimaging procedures. Their postnatal neurodevelopment leads to more complex synaptic organization, allowing better behavioral characterization [4].

The high failure rates of drug research and development for AD have raised questions about the therapeutic strategies used, which were initially focused on symptomatic treatment as cognitive enhancement. Later, anti-Aβ agents were successfully tested in preclinical models but failed in clinical studies owing to the poor translation between preclinical and clinical research [9, 10]. Together with the use of proper animal models, the translation to the clinic can be improved if the same techniques are applied to the study of AD progression in humans and animals. Along this line, neuroimaging techniques represent an excellent approach because they can be applied to both experimental and clinical studies [7]. Magnetic resonance imaging (MRI) has been shown to be useful in studying AD biomarkers [11–13], including volumetric studies to evaluate atrophy based on structural MRI [14, 15], assessment of tissue changes in gray matter (GM) or white matter (WM) by diffusion-weighted MRI (DWI) [16, 17], or analysis of structural and functional disconnection associated with both prodromal and dementia stages of the disease [18–23]. These MRI studies suggest that the cognitive decline in AD may be related to functional or structural disconnection between brain regions rather than to changes localized in isolated brain areas [24–26].

The analysis of brain networks by means of graph theory has been widely used in recent years to characterize structural and functional connectivity in healthy or pathological brains [27, 28]. By using a connectomics approach, network properties can be measured to characterize the network and its disease-associated alterations. These properties describe network integration, which is the network’s ability to efficiently transfer and combine information among the different regions [29], and network segregation, which is related to the presence of clusters of densely interconnected brain regions for specialized processing [29]. The network definition is based on nodes (regions that are connected) and links (connections between regions) that are usually extracted from different modalities of MRI, including structural T2-weighted, DWI, and functional MRI [30]. In patients with AD, alterations in functional brain networks [31] and in structural networks have been described on the basis of common GM patterns [32] or WM tracts [18]. Analysis of GM networks has shown less efficient topological structure in AD than in healthy controls. In mild cognitive impairment (MCI), which can evolve to AD, research has shown intermediate topological properties between healthy persons and subjects with AD [32–34]. A decrease in network efficiency in patients with AD was also observed in diffusion-based connectomes [18, 21, 35], in addition to impairment in functional network properties assessed by magnetoencephalography [36], electroencephalography [37], or functional MRI [31, 38]. MRI-based connectomics can be applied to animal models, allowing for characterization of early stages of the disease. Moreover, using an animal model of vascular cognitive impairment (VCI), our group demonstrated that network-based structural connectomics reveals neuronal alterations before the onset of executive function impairment [39]. Network metrics correlated very well with behavioral results, discriminating with high accuracy spontaneous hypertensive from normotensive rats. Indeed, on the basis of this network analysis, our group was able to predict the level of future cognitive impairment in our experimental model, supporting the hypothesis that DWI-based connectomics and subsequent global network analysis were an appropriate noninvasive imaging biomarker of VCI and probably other neurodegenerative disorders as well. However, although some researchers have described alterations in connectivity associated with AD in other animal models [40, 41], to the best of our knowledge, there are no studies describing the changes in the brain network at a global level by means of graph theory in rat models of AD and these changes’ association with cognitive decline.

In this work, we aimed to characterize the brain structural and functional network of the TgF344-AD rat model at an early period, before appreciable Aβ plaque formation [8], and to correlate the network features with impairments in learning and working memory performance. A trend toward worse performance of these rats in the Barnes maze reversal phase was described previously [8], indicating impaired spatial reference memory as early as 6 months of age. In this study, we evaluated cognitive performance at an earlier stage, starting with a training phase at 2 months of age, to identify the relationship between cognitive outcome and brain network organization. Alterations in global and regional structural networks were found, together with regional differences in functional networks. These differences were similar to the ones reported in human cohorts evaluated at more advanced stages of the disease. These results point to the translational possibilities of this kind of analysis to contribute to the definition of early biomarkers of AD and to more efficient development of new therapeutic drugs against the disease.

## Methods

### Animals

The experiments were carried out using a cohort of 18 male rats, including 9 transgenic TgF344-AD rats [8] and 9 wild-type Fischer rats. Rats were housed in cages under controlled temperature (21 ± 1 °C) and humidity (55 ± 10%) with a 12-h light/12-h dark cycle (light between 8:00 a.m. and 8:00 p.m.). Food and water were available ad libitum during all experiments, except during the period of behavioral training and testing, when they received only 75% of their usual food intake. Animal work was performed in accordance with the local legislation (Decret 214/1997 of July 20 by the Departament d’Agricultura, Ramaderia i Pesca de la Generalitat de Catalunya), with the approval of the Experimental Animal Ethical Committee of the University of Barcelona, and in compliance with European legislation.

### Cognitive function evaluation

Working memory performance was evaluated by means of the delayed nonmatch-to-sample (DNMS) task following a procedure slightly modified from a previous study [42] by using isolated operant chambers (Med Associates, Fairfax, VT, USA). The chambers have a pellet dispenser and three retractile levers, two of them in the same chamber side where the feeder is (namely, right and left levers) and the other in the opposite side of the chamber (center lever). During the behavioral testing weeks, rats were food-deprived, receiving only 75% of their usual food intake. Animals underwent a habituation period and six training phases before the start of the DNMS task itself, all of which is explained in detail in Additional file 1. In short, training stages 5 and 6 are similar to the DNMS protocol, explained below, but with no delay between levers at stage 5 and a random delay of 1 to 5 s between the lever exposures at stage 6. In both cases, training stage is repeated until the animal has 2 consecutive days with a score of a minimum of 80% correct responses. The number of sessions required to reach the criteria was recorded.

Once an animal achieved the acquisition criteria, the DNMS task began. This task required the animal to press the retractable lever presented on a random basis on the left or right (sample response) to initiate the trial. This began a delay phase randomly timed between 1 and 30 s. After the delay, the animal had to press the center lever, located on the opposite wall. The animal then returned to where both the left and right levers were extended (match/nonmatch phase). The correct response required a press on the lever opposite the one pressed during the sample phase (constituting the nonmatch response), which was followed by delivery of a food pellet into the hopper. An incorrect response (pressing the same lever as the one pressed in the sample phase) produced a 5-s time-out in which the overhead lights were turned off and no sucrose pellet was delivered. Trials were separated by 10 s. Each session finished after 90 min or when 90 trials had been completed.

The animals performed this protocol during 15 sessions (5 sessions per week). The number of trials and the percentage of correct responses by the animals in each session were recorded, taking into account the different delays between samples.

### Magnetic resonance imaging

MRI experiments were conducted on a 7.0-T BioSpec 70/30 horizontal animal scanner (Bruker BioSpin, Ettlingen, Germany) equipped with an actively shielded gradient system (400 mT/m, 12-cm inner diameter). The receiver coil was a four-channel phased-array surface coil for the rat brain. Animals were placed in supine position in a Plexiglas holder with a nose cone for administration of anesthetic gases (1.5% isoflurane in a mixture of 30% O_2_ and 70% CO) and were fixed using a tooth bar, ear bars, and adhesive tape. Then, the rat received a 0.5-ml bolus of medetomidine (0.05 mg/kg subcutaneously), and a catheter was implanted in the back of the rat for continuous perfusion of medetomidine during the experiment. Isoflurane was gradually decreased until reaching 0%, and 15 min after the bolus was delivered, the perfusion of medetomidine (0.05 mg/kg subcutaneously) started at a rate of 1 ml/h.

3D localizer scans were used to ensure accurate position of the head at the isocenter of the magnet. T2-weighted images were acquired by a rapid acquisition with relaxation enhancement (RARE) sequence with an effective echo time (TE) of 35.3 milliseconds, repetition time (TR) of 6000 milliseconds, and RARE factor of 8. Matrix size was 256 × 256 with an in-plane voxel size of 0.12 × 0.12 mm^2^, 40 slices, and slice thickness of 0.8 mm, resulting in a field of view (FOV) of 30 × 30 × 32 mm^3^. T1-weighted images were acquired using a modified driven equilibrium transform (MDEFT) protocol with TE = 2 milliseconds, TR = 4000 milliseconds, matrix size: 256 × 256 × 36, and voxel size 0.14 × 0.14 × 0.5 mm^3^, resulting in an FOV of 35 × 35 × 18 mm^3^.

DWI scans were acquired using a spin echo planar imaging (EPI) sequence (TE = 24.86 milliseconds, TR = 15,000 milliseconds, 4 segments) with 60 gradient directions with *b* value 1000 s/mm^2^ and 5 volumes without diffusion weight (*b* value = 0 s/mm^2^). Sixty slices were acquired with a matrix size of 72 × 72 and an isometric voxel size of 0.31 × 0.31 × 0.31 mm^3^, resulting in an FOV of 22.23 × 22.23 × 18.54 mm^3^. Resting-state functional MRI (rs-fMRI) scans were acquired using a T2*-weighted acquisition (TE = 10.75 milliseconds, TR = 2000 milliseconds) and 600 volumes (20 min). Thirty-four slices were acquired with a matrix size of 64 × 64 and voxel size of 0.4 × 0.4 × 0.6 mm^3^. The FOV was 25.6 × 25.6 × 20.4 mm^3^.

### Image processing

The acquired images were processed to obtain both structural and functional connectomes. T1-weighted and T2-weighted images were used for tissue segmentation and parcellation respectively, because of their high resolution and tissue contrast. Diffusion-weighted volumes were used to estimate the structural connectivity, and rs-fMRI was performed to assess the functional connections.

#### Anatomical images: skull stripping, tissue segmentation, and parcellation

A rat brain atlas, including a T2-weighted template, brain mask, tissue probability maps (TPMs), and region parcellation, was used for skull stripping and for tissue segmentation and parcellation. This atlas was a combination of atlases published previously [43, 44]. On one hand, the former provides a whole-brain parcellation based on the Paxinos and Watson rat brain atlas [45] but does not specify WM, GM, and cerebrospinal fluid (CSF) TPMs required to segment the brain in these three kinds of tissues. On the other hand, the second atlas [44] provides the TPMs of these tissues, but parcellation is constrained to cortical regions instead of the whole brain. For this reason, a combination of both atlases was used. The template in the publication by Schwarz et al. [43] was registered to the template in the publication by Valdés-Hernández et al. [44] using the elastic diffeomorphic registration algorithm implemented using advanced normalization tools (ANTs) [46]. The resulting transformation was applied to the label volume to align the whole-brain parcellation with the template provided by Valdés-Hernández et al. [44].

For each animal’s acquisition, first the T2-weighted image was denoised using a nonlocal means denoising filter [47], and then intensity bias was corrected with the N4ITK algorithm [48]. Afterward, the atlas template was registered to the preprocessed T2-weighted volume by elastic registration [46], and the resulting transformation was applied to the brain mask to skull-strip the subject volume. TPMs and regional labels were translated to the rat’s T2-weighted image on the basis of registration between the atlas template and the masked brain.

T1-weighted MDEFT volume was used for tissue segmentation, owing to the high contrast between WM and GM observed in this modality. For each rat, brain mask and TPMs were propagated from the T2-weighted image to the T1-weighted image using affine registration. Segmentation was performed by using the unified segmentation model [49] available in SPM software (SPM8 release; www.fil.ion.ucl.ac.uk/spm) using these TPMs as a priori maps. The resulting segmentation and TPMs were transformed back to the T2-weighted images.

#### DWI image processing

Diffusion-weighted volumes were preprocessed, including eddy current correction using FSL [50], denoising [47], and bias correction [48]. The five non-diffusion-weighted images (baseline images) were averaged and registered to the T2-weighted image to correct for spin EPI distortions. Then, brain mask, region maps, and TPMs were translated from the T2-weighted images to the preprocessed diffusion images. A diffusion tensor model was fitted to the images using Dipy [51], and fractional anisotropy (FA) was computed. Tractography was performed using a deterministic algorithm based on constrained spherical deconvolution model, considering as seed points for streamlines the voxels comprised in the WM mask obtained from segmentation and FA < 0.1 as a stop criterion.

#### Resting-state functional MRI processing

Resting-state preprocessing included slice timing, motion correction by spatial realignment using SPM8, and correction of spin EPI distortion by elastic registration to the T2-weighted volume using ANTs [46]. Brain mask, region parcellation, and TPMs were registered from T2-weighted volume to the preprocessed mean resting-state volume. The whole-brain mask was applied to skull-strip the brain in the resting-state volumes, removing voxels that did not correspond to brain tissue. Further processing was performed only over the extracted brain: z-score normalization and detrending of the time series, smoothing with an FWHM of 1.2 mm, frequency filtering of the time series between 0.01 and 0.1 Hz, and regression by motion parameters and WM and CSF average signal. All these steps were performed using NiTime (http://nipy.org/nitime/).

### Connectome construction

#### Structural connectome

Brain regions identified by parcellation were considered as nodes of the brain network. The original parcellation published previously [43] consisted of 101 regions. However, owing to the lower resolution of the acquired diffusion images, some of the smallest regions could not be accurately identified, so a reduced version of 76 regions resulting from combination of the original areas was considered. A list of the regions is provided in Table 1.

**Table 1:** Brain regions included in structural and functional connectomes

Region	Structural connectome		Functional connectome	
	Right	Left	Right	Left
Accumbens	1	39	1	28
Amygdala	2	40	2	29
Anterior commissure and bed nucleus	3	41	–	–
Caudate putamen and globus pallidus	4	42	3	30
Corpus callosum	5	43	–	–
Auditory cortex	6	44	4	31
Cingulate cortex	7	45	5	32
Entorhinal cortex	8	46	6	33
Frontal association cortex	9	47	7	34
Insular cortex	10	48	8	35
Medial prefrontal cortex	11	49	9	36
Motor cortex	12	50	10	37
Orbitofrontal cortex	13	51	11	38
Parietal association and somatosensory cortex	14	52	12	39
Piriform cortex	15	53	13	40
Retrosplenial cortex	16	54	14	41
Temporal association cortex	17	55	15	42
Visual cortex	18	56	16	43
Septum and diagonal band	19	57	17	44
Hippocampus anterodorsal	20	58	18	45
Hippocampus posterior	21	59	19	46
Hippocampus subiculum	22	60	20	47
Hippocampus ventral and hypothalamus lateral	23	61	21	48
Hypothalamus medial and ventral tegmental area	24	62	22	49
Internal capsule	25	63	–	–
Interstitial nucleus of the posterior limb of the anterior commissure, olfactory nuclei, substantia innominata, and ventral pallidum	26	64	23	50
Medial geniculate	27	65	–	–
Mesencephalic region	28	66	–	–
Olfactory tubercle	29	67	24	51
Periaqueductal gray	30	68	–	–
Pons	31	69	–	–
Raphe	32	70	–	–
Substantia nigra	33	71	–	–
Superior colliculus	34	72	25	52
Thalamus dorsolateral	35	73	26	53
Thalamus midline dorsal and ventromedial	36	74	27	54
Zona incerta	37	75	–	–
Fimbria	38	76	–	–

Region name based on previous publication [42] and right and left hemisphere region indexes in the structural and/or functional connectomes

A connection between two regions, *I* and *J*, was defined if there was at least one streamline with a starting point in *I* and ending in *J*. Two different connection weights were considered: (1) the average FA in all the streamlines connecting each pair of regions and (2) the fiber density (FD) of the connection defined as in a previous publication [52] as follows:

1 $$ \mathrm{FD}=\frac{1}{V_i\cdot {V}_j}\cdot \sum \limits_{s\in {S}_{i,j}}\frac{1}{l(s)} $$

where *S*_*ij*_ is the set of streamlines connecting regions *I* and *J*, *l(s)* is the length of the streamline *s*, and *V*_*i*_ and *V*_*j*_ are the volumes of regions *I* and *J*, respectively. In addition to the FA-weighted connectome (FA-w) and the FD-weighted connectome (FD-w), a binary connectome was considered, where a value of 1 in the connection matrix indicated a connection between two regions and otherwise the value was 0.

#### Functional connectome

Network nodes were defined from the regions obtained by parcellation. As in the case of diffusion images, resting-state functional volumes have lower resolution than T2-weighted images, and not all the regions could be identified reliably, so small regions were combined into bigger ones, which resulted in the same 76 regions considered in the structural connectome. However, because brain activity is constrained to GM areas [53], regions comprising only WM tissue were not considered, which resulted in the definition of 54 regions as network nodes, shown in Table 1. The preprocessed time series were averaged at each region, allowing us to obtain the regional time series.

The weight of each connection was defined as the partial correlation coefficient between each pair of regional time series, transformed by Fisher’s *z*-transformation. Negative correlation coefficients were excluded. A binary functional connectome was obtained by setting to 1 connections where *z* > 0, and 0 otherwise.

### Brain network analysis

Graph theory metrics were used to describe the network organization at a global and regional level. They included basic measures such as the number and weight of connections, as well as measures of functional integration and segregation [53].

Degree and strength are basic measures of the amount of connectivity. For a given node, the nodal degree is the number of nodes connected to it, and the nodal strength is the sum of the weights of all its connections. Global degree and strength are the result of averaging the nodal degree or strength, respectively, of all the nodes in the brain. A higher degree or strength indicates more or stronger connections at a global level, which could be related to shorter path lengths to transmit information between nodes.

On one hand, integration has been associated with the ability to rapidly combine specialized information from the distributed brain regions at a global level [53]. Integration measures are based on the shortest path length, that is, the minimum distance between two nodes. At a global level, we considered the global efficiency, which is inversely related to the path length. High values of global efficiencies are related to short distances between network nodes, allowing for fast communication between pairs of brain regions.

On the other hand, segregation is the ability of specialized processing to occur within densely interconnected groups of brain regions. Thus, nodal efficiency measures the efficiency of the subnetwork associated with a given node, and the nodal clustering coefficient measures the number of neighbors of a given node that are also neighbors of each other. These regional (nodal) metrics can be averaged in the whole brain to obtain the local efficiency and average clustering coefficient. High local efficiency and clustering are related to both a highly segregated network and a high number or weight of connections.

### Statistics

Kruskal-Wallis tests were used to evaluate statistical significance between groups. In order to take into account the influence of the age of the rats in the network metrics, a generalized linear model with rat age as a covariate was used to evaluate the statistical significance of the differences in network metrics between the two groups. Multiple comparisons correction in the analysis of regional network metrics was performed using the false discovery rate (FDR) [54]. Spearman’s correlation coefficient was computed to evaluate the relationship between cognitive performance and network metrics.

## Results

### Cognitive function evaluation

**Fig. 1:**
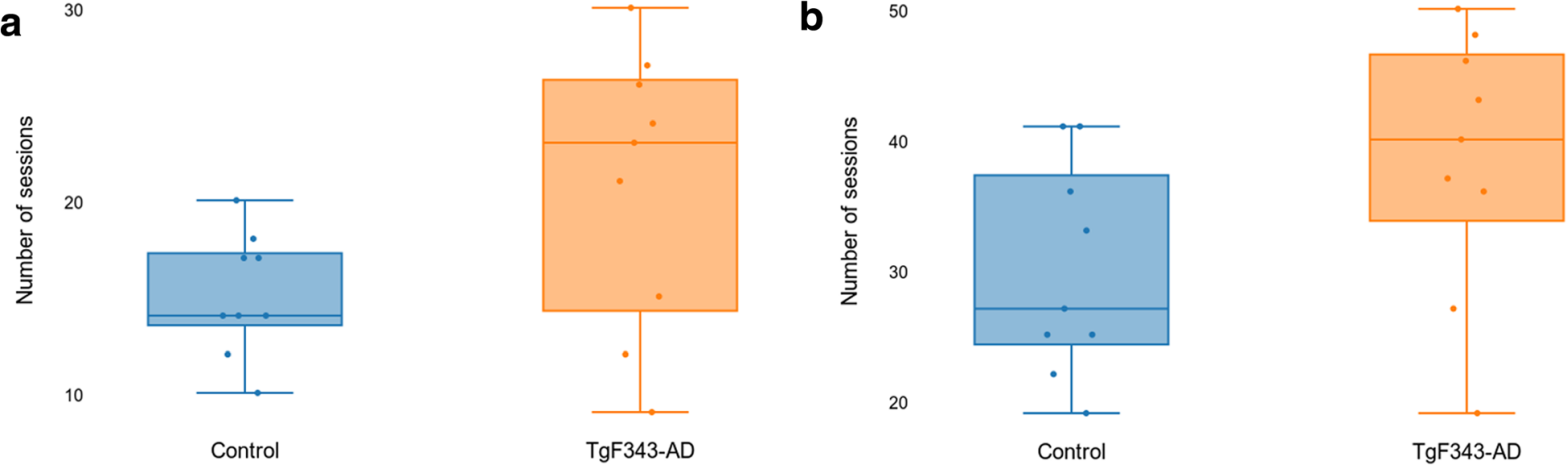
Number of training sessions required to achieve the acquisition criteria. **a** Number of sessions required to achieve training stage 5. **b** Total number of training sessions required to achieve the criteria. Each dot corresponds to the number of sessions performed by one of the rats

Cognitive function was evaluated in two different phases: the training phase, more related to learning processes, and the DNMS phase, specifically related to working memory. In the training phase, both the number of sessions required to achieve training stage 5 (similar to the DNMS task but without delays between levers) and the total number of sessions required to reach the DNMS phase were moderately higher in the transgenic animals than in the controls, although no significant differences were found (*p* = 0.0764 and *p* = 0.0695, respectively) (Fig. 1).

**Fig. 2:**
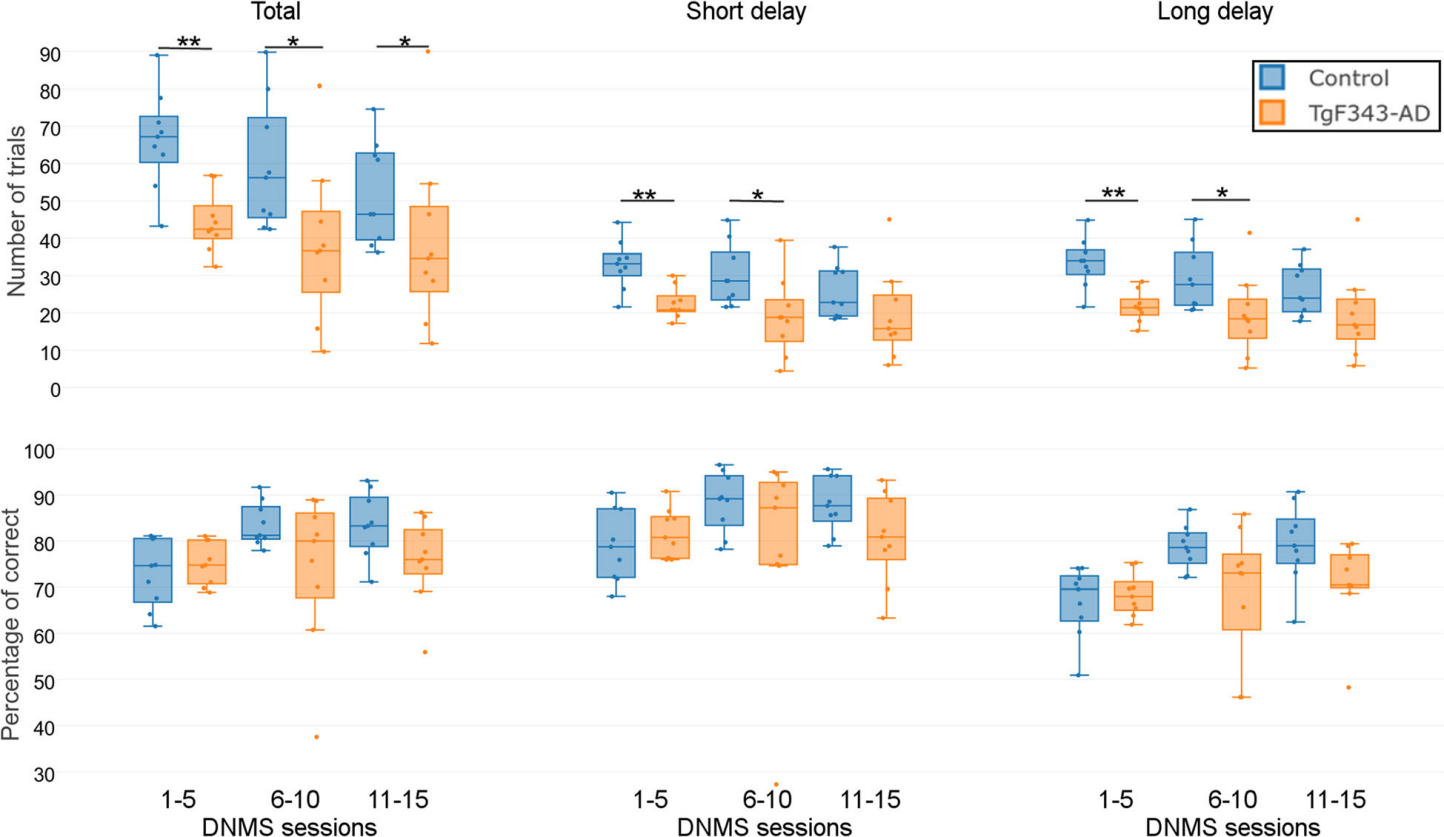
Performance in the delayed nonmatch to sample (DNMS) task. Number of trials (*top*) and percentage of correct responses (*bottom*), considering all trials (total, *first column*), trials where the random delay was less than 15 s (short delay, *second column*), and trials where the random delay was more than 15 s (long delay, *third column*). *Blue* corresponds to controls and *orange* to transgenic rats. Significant differences between groups are indicated: * *p* < 0.05 and ** *p* < 0.01

Regarding the DNMS phase, the number of trials and the percentage of correct responses were considered. As shown in Fig. 2, the number of trials was significantly higher in control rats than in the transgenic rats. This difference was consistent if the total number of trials was considered (*p* = 0.0152) or if short-delay (*p* = 0.0152) and long-delay (*p* = 0.0118) trials were evaluated separately. Of note, the differences were bigger in the first five sessions (first week) of testing (total trials, *p* = 0.0023; short delay, *p* = 0.0022; long delay, *p* = 0.0014), whereas significance decreased as the rats underwent more testing sessions (second week total trials, *p* = 0.0192; short delay, *p* = 0.0150; long delay, *p* = 0.0216; third week total trials, *p* = 0.0464; short delay, nonsignificant; long delay, nonsignificant). The percentage of correct responses was similar in both genotypes, shown in Fig. 2.

As a consequence of the differences in the training period, there are differences in the ages of the animals when they finished the DNMS task. On average, control animals finished the task at 151.11 ± 12.06 days of age (ranging from 138 to 169 days), whereas TgF344-AD rats finished the DNMS sessions when they were 181 ± 32.6 days old (ranging from 139 to 225 days). The MRI acquisition of each of the animals was performed the day after they finished the DNMS period.

### Structural connectome

**Fig. 3:**
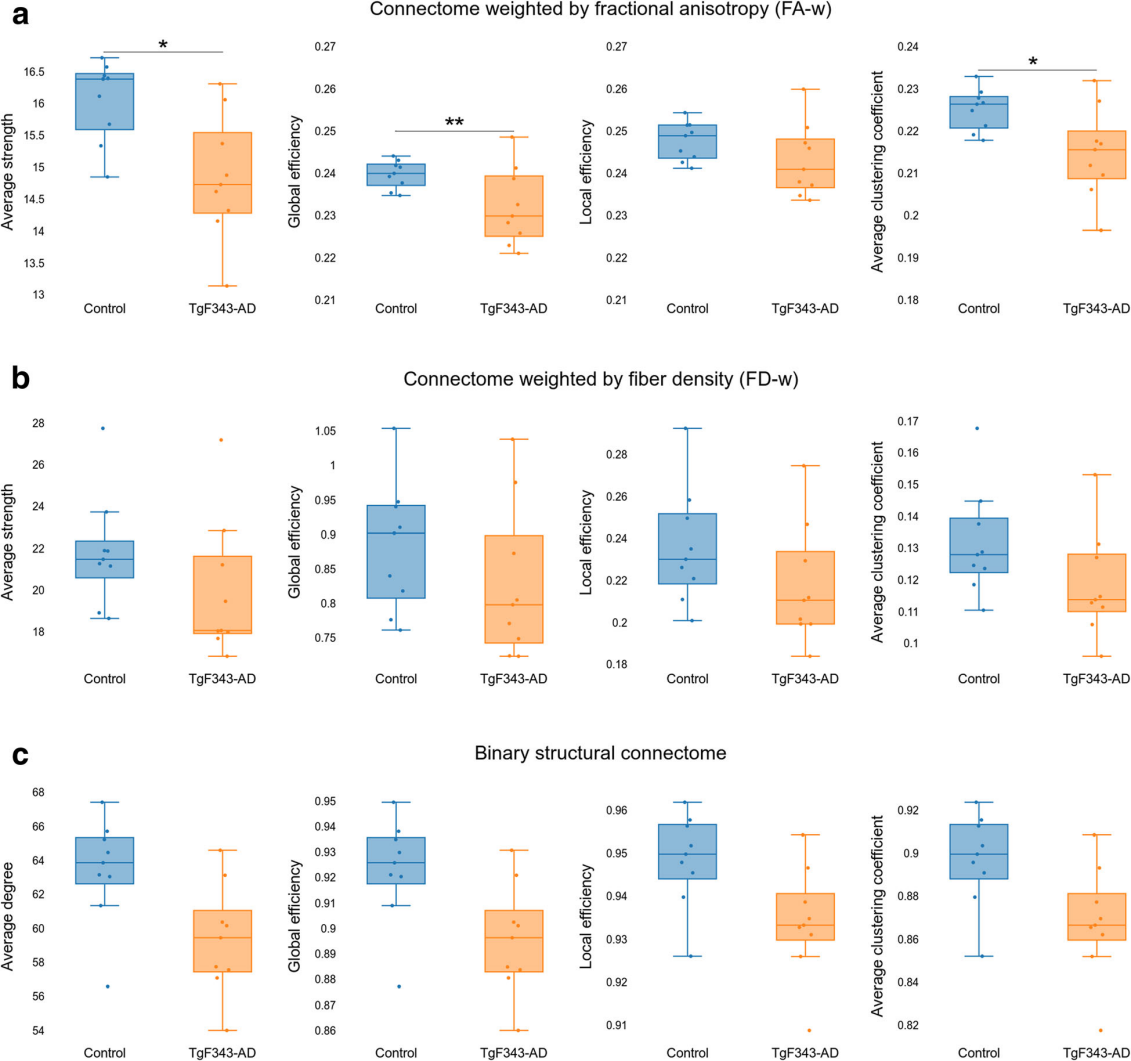
Global network metrics of the structural connectomes. **a** Global metrics of the connectome weighted by fractional anisotropy (FA-w). **b** Global metrics of the connectome weighted by fiber density (FD-w). **c** Global metrics of the binary structural connectome. Significant differences between groups are indicated: * *p* < 0.05 and ** *p* < 0.01

Figure 3 shows the global network parameters of FA-w, FD-w, and binary connectomes, where a trend of AD animals toward lower network metrics than in control animals was observed. Statistically significant differences were found in the FA-w connectome average strength (*p* = 0.0405), global efficiency (*p* = 0.0039), and average clustering coefficient (*p* = 0.0403).

**Fig. 4:**
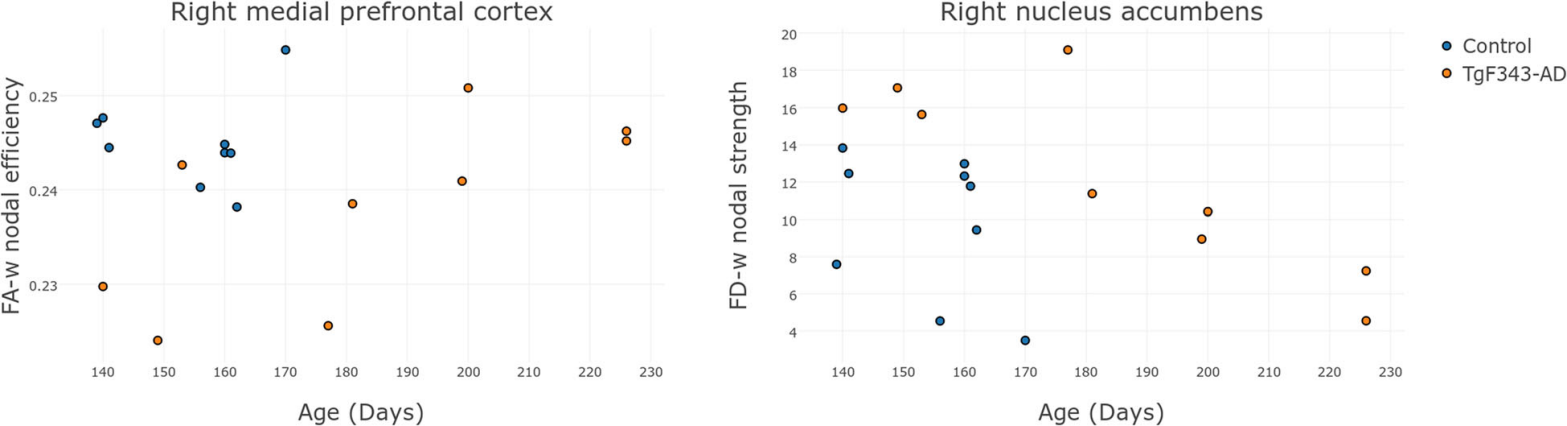
Regional network metrics of the structural connectomes. Structural regional network metrics in the anatomic regions where statistically significant differences (false discovery rate-corrected *p* < 0.05) were observed between control and transgenic rats. *FA-w* Connectome weighted by fractional anisotropy, *FD-w* Connectome weighted by fiber density

On one hand, regarding regional differences, the right medial prefrontal cortex showed decreased FA-w nodal efficiency in the AD group compared with the controls (FDR-corrected *p* value = 0.0409). On the other hand, the right nucleus accumbens was characterized by a higher FD-w strength in the transgenic group than in the controls (FDR-corrected *p* value = 0.0294). Figure 4 shows the distribution of these network metric values in these regions with respect to age in control and transgenic rats.

### Functional connectome

**Fig. 5:**
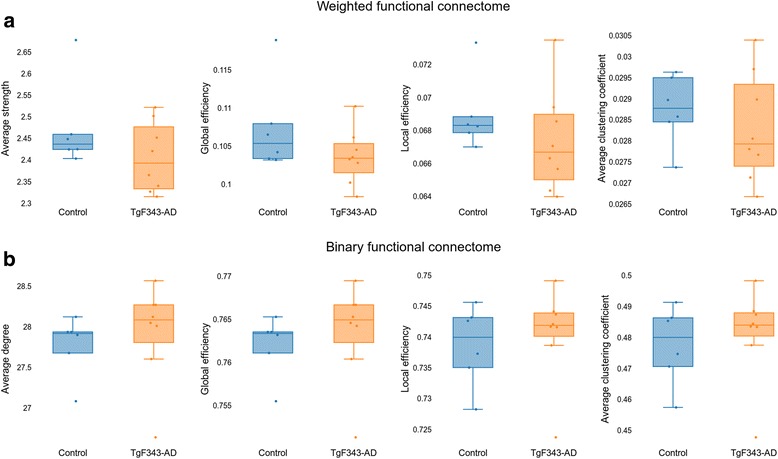
Global network metrics of the functional connectomes. **a** Global metrics of the weighted functional connectome. **b** Global metrics of the binary functional connectome. No statistically significant differences between groups were found

**Fig. 6:**
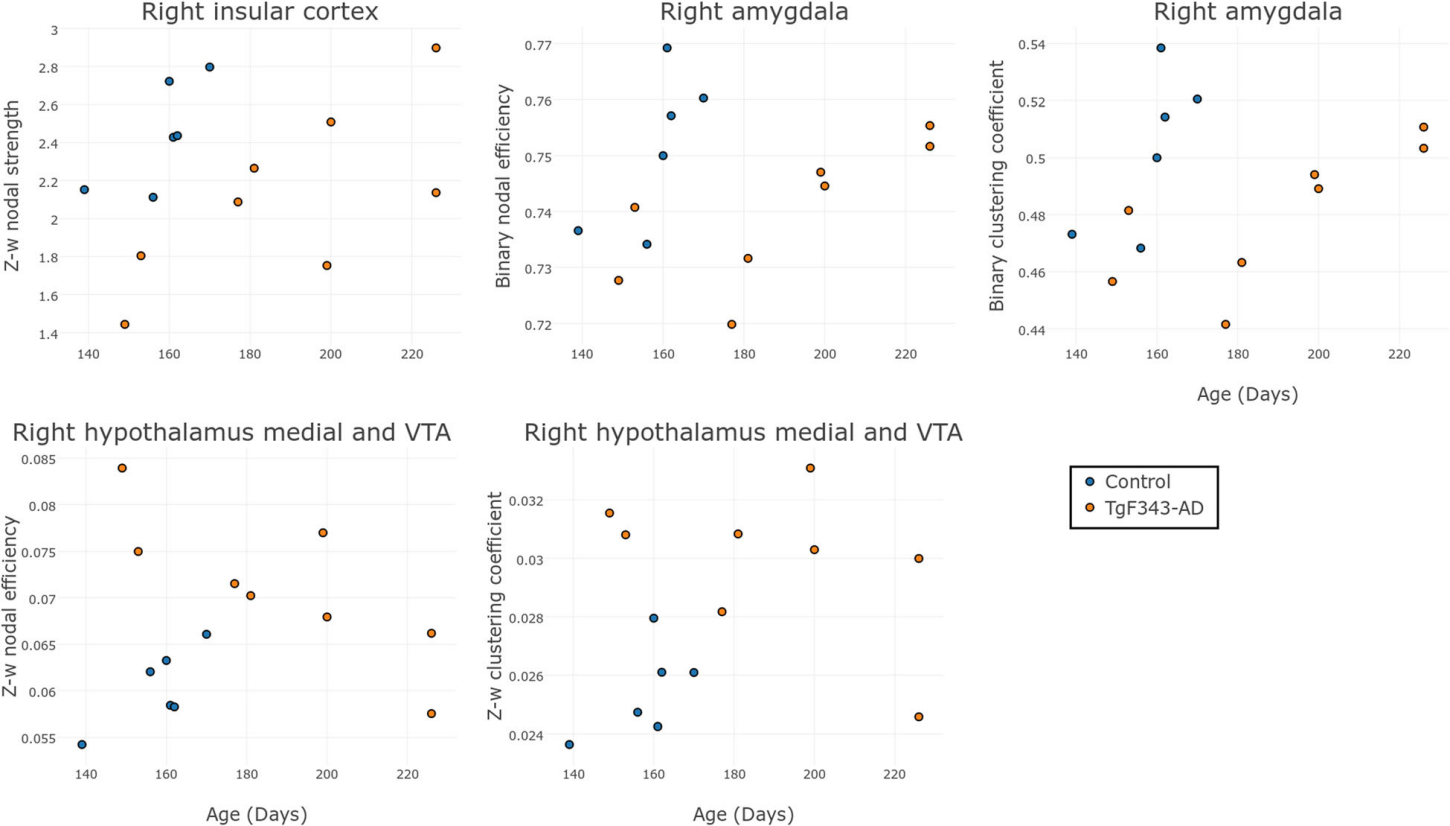
Regional network metrics of the functional connectomes. Functional regional network metrics in the anatomic regions where statistically significant differences (FDR-corrected *p* < 0.05) were observed between control and transgenic rats

Figure 5 shows the global metrics obtained from the functional binary and weighted connectome, where no significant differences were observed in any of the studied metrics. Interestingly, differences between genotypes were found at the regional level (Fig. 6). Nodal strength in the right insular cortex was higher in controls than in AD animals (FDR-corrected *p* = 0.0157). Significant differences were also found in weighted nodal efficiency (FDR-corrected *p* = 0.0004) and clustering coefficient (FDR-corrected *p* = 0.0110) of the right hypothalamus medial tegmental area and ventral tegmental area (VTA), where the transgenic group showed higher values of the two metrics than the control cohort. The binary nodal efficiency (FDR-corrected *p* = 0.0449) and clustering coefficient (FDR-corrected *p* = 0.0475) of the right amygdala were significantly higher in control than in transgenic rats. As shown in Fig. 6, there was also an age-related dependence in these measurements. It can be also noted that only TgF344-AD rats were scanned later than 170 days, because this group required a longer training period than control animals.

### Correlation between cognitive function and network metrics

The relationship between global and regional network metrics and the performance of the animals in both cognitive training and DNMS phases was evaluated. Strong correlations (Spearman’s correlation coefficient |*r*| > 0.7) are reported and explained below.

#### Training phase: learning evaluation

**Fig. 7:**
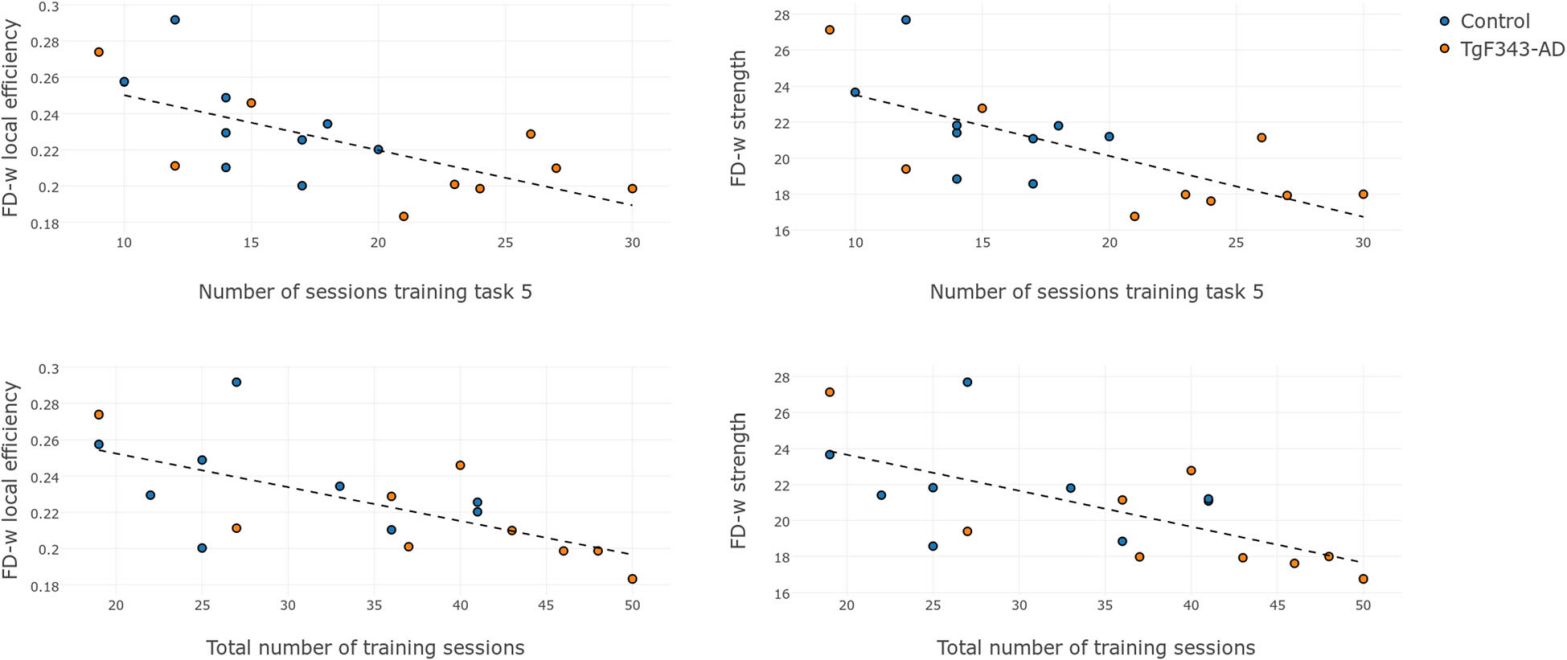
Correlation between training sessions and network metrics. Relationship between FD-w local efficiency and strength with the number of sessions required to achieve the acquisition criteria in training stage 5 (*first row*) and the total number of training sessions (*second row*) in control and transgenic animals. *FD-w* Connectome weighted by fiber density

The FD-w local efficiency and strength were negatively correlated with the number of sessions required to achieve the criteria in training stage 5 (*r* = − 0.7029, *r* = − 0.7226, respectively). As explained above, this stage is similar to the DNMS task, but with no delays between levers exposure. These metrics were also correlated with the total number of training sessions (*r* = − 0.7015, *r* = − 0.7046), as shown in Fig. 7. However, no strong correlation between the number of training sessions and the global functional network metrics was observed (data not shown).

Performance in the training protocol was also highly correlated with some regional metrics, as summarized in Table 2.

**Table 2:** Correlation between required training sessions and regional network metrics

	Region	Connectome	Metric	*r* Value
Total training sessions	L Ent Cx	FD-w	NE	−0.7708
			CC	−0.7450
	R Pir Cx	Binary functional	NE	0.7122
			CC	0.7122
	R TeA Cx	FD-w	NE	−0.7056
	L IPAC, olfactory nuclei, SI, and VP	FD-w	NE	− 0.7418
	R Tu	Binary functional	NE	0.7387
			CC	0.7387
	L Tu	FA-w	Strength	−0.7708
	L Pons	FD-w	CC	−0.7284
Training stage 4	L VHc and LH	Binary functional	Degree	0.7049
Training stage 5	R Amygdala	Weighted functional	Strength	−0.7797
	L Ent Cx	FD-w	NE	−0.9027
			CC	−0.8944
			Strength	−0.8333
	R Pir Cx	FD-w	NE	−0.7329
			CC	−0.7484
			Strength	−0.7070
	L RS Cx	FD-w	Strength	−0.7412
	R VCx	FD-w	NE	−0.7412
	L ADHc	Binary functional	Degree	−0.7137
	L IPAC, olfactory nuclei, SI, and VP	FD-w	NE	−0.8260
			CC	−0.7450
	Left olfactory tubercle	FA-w	Strength	−0.7091
Training stage 6	R Amygdala	Binary functional	Degree	−0.7122
	L RS Cx	Weighted functional	Strength	−0.7024
	L Septum and DB	Weighted functional	NE	−0.8049

*Abbreviations: NE* Nodal efficiency, *CC* Clustering coefficient, *Cx* Cortex, *L* Left, *R* Right, *Ent Cx* Entorhinal cortex, *Pir CX* Piriform cortex, *TeA Cx* Temporal association cortex, *IPAC* Interstitial nucleus of the posterior limb of the anterior commissure, *SI* Substantia innominata, *VP* Ventral pallidum, *Tu* Olfactory tubercle, *VHc* Ventral hippocampus, *LH* Lateral hypothalamus, *RS* Retrosplenial cortex, *VCx* Visual cortex, *ADHc* Anterodorsal hippocampus, *DB* Diagonal band, *FA-w* Connectome weighted by fractional anisotropy, *FD-w* Connectome weighted by fiber density

Spearman’s correlation coefficient (*r*) between the number of sessions required to achieve the criteria in training stages and the regional network metrics of the structural and functional connectomes

#### DNMS phase: working memory evaluation

Global network metrics were not strongly correlated with performance in the DNMS phase (|*r*| < 0.7). Nevertheless, some regions were identified as having functional and structural network metrics highly correlated with the number of trials carried out by the animal in the DNMS phase. Specifically, the left entorhinal cortex FD-w clustering coefficient was positively correlated with the total number of trials, taking into account either all (*r* = 0.7090) or only short (*r* = 0.7131) delays, whereas the left interstitial nucleus of the posterior limb of the anterior commissure FD-w clustering coefficient was highly correlated only with the number of trials with short delay (*r* = 0.7077). Regarding the functional network metrics, the binary nodal efficiency and clustering coefficient of the right piriform cortex were negatively correlated with the number of trials with long delays performed by the animal (*r* = − 0.7028). The percentage of correct responses showed correlations < 0.7 for all studied network metrics.

## Discussion

Early diagnosis of AD is essential for the development of treatments. In this sense, the use of animal models allows for longitudinal follow-up and can contribute to the understanding of the mechanisms underlying AD preclinical stage [4]. MRI has been shown to be useful as a modality for detecting early AD biomarkers [11, 12]. In addition, it represents a highly translational method because it can be applied to both animal model research and human studies, allowing for comparable results [10]. Researchers in MRI-based studies have reported evidence supporting the hypothesis of AD as a disconnection syndrome; that is, functional and structural interactions between brain regions instead of alterations in isolated brain areas could be responsible for the cognitive impairment in AD [24, 26]. Therefore, in the present study, we evaluated connectivity in an animal model of AD based on MRI evaluation at an early stage to contribute to the characterization of AD early stages using a translational approach.

### Cognitive impairment

The TgF344-AD model used in this study replicates all the pathological hallmarks of AD in an age-dependent manner [8]. TgF344-AD rats exhibit progressive cognitive impairment, including hyperactivity and abnormalities in open field activity and spatial learning and memory. Authors of previous works reported significant cognitive impairment in aged rats of 15 months of age [8] and visual functional impairment (lower visual acuity) at 10 months of age [55]. At earlier periods, significant differences have not been described, although a trend toward impaired memory at 6 months of age was shown [8], as revealed by more errors in the reversal phase of the Barnes maze test [56]. Likewise, most of the AD pathological hallmarks, such as neuronal loss and Aβ plaque formation, are still not significant at this early age, although tau changes have already been detected [8]. In our study, rats started the training phase for the DNMS protocol at 2 months of age. To the best of our knowledge, cognitive function in this model had not previously been evaluated at such an early period. Differences in learning abilities of a memory-related task were already present at this age, resulting in longer training periods required for AD rats to meet the acquisition criteria. Along this line, learning disabilities as well as spatial memory impairment have previously been reported in a different transgenic rat model (McGill-R-Thy1-AAP Tg), also at 3 months of age [5, 6], with use of the Morris water maze task [57], which also involves spatial learning and memory.

After reaching the final training criteria, both groups showed similar percentages of correct responses. Although this trend was maintained during the DNMS task, the number of trials completed by transgenic rats was significantly less than the number of trials completed by control rats. As the number of sessions increased, the number of trials decreased in the same manner in both genotypes. This is logical because they increased their percentage of correct responses, optimizing the number of trials and leading more quickly to a satiety point. A loss of motivation could also explain this behavior because a decrease in both the number of trials and the number of correct responses was observed in stage 6 of the training phase (Additional file 1: Figure S1a), a finding more evident for AD rats than for control animals. We hypothesize that if control animals required fewer sessions to finish the training, their motivation during the DNMS phase might be higher than that of AD rats, and therefore they might be able to perform more trials. Indeed, the number of training sessions required was highly correlated with the number of trials in the testing phase (Additional file 1: Figure S1b). An anxiety-like phenotype has been described in the McGill-R-Thy1-AAP transgenic rat from adulthood (6 months old) to middle age (12 months old) [5]. Although it was not evaluated in this study, anxiety might also contribute to the lower number of trials performed by AD rats. In agreement with our results, learning and spatial reference memory impairments were also demonstrated in the McGill-R-Thy1-AAP rats at the preplaque stage (3 months of age) [58].

### Structural connectivity alterations

The results of the structural connectome analysis indicated different organization of the whole-brain network in control and transgenic rats. Although statistically significant differences were assessed only in the FA-w connectome, AD animals were characterized by a decrease in global and local efficiency, clustering, and strength in comparison with control animals. These findings are coherent with the network parameters evaluated in human cohorts at more advanced stages of the disease, where patients with AD showed significantly reduced global efficiency in comparison with age- and sex-matched control subjects [21]. A decrease in degree and efficiency in the AD subjects consistent among the different levels of the pathology was described in a study based on the core network [18]. Likewise, decreased global efficiency, strength, degree, and clustering coefficient of the fiber number-weighted network were shown in patients with different types of MCI or preclinical AD [19, 35, 59–61], as well as decreased local efficiency in patients with early AD [62]. In populations with a genetic risk of AD, such as carriers of the *apolipoprotein E* (*APOE*) *ε4* and *rs405509* alleles, decreased global and local efficiency of FA-w networks have been described [63, 64]. These metrics have also been used to predict dementia, as described elsewhere [65].

In TgF344-AD and control animals, regional differences in the structural connectome were found in the right medial prefrontal cortex (as decreased FA-w nodal efficiency in AD). Accordingly, studies in human populations have revealed reduced nodal efficiency in medial frontal cortical structures of the right hemisphere in AD compared with healthy subjects [21]. Fiber number-weighted nodal efficiency and/or strength was also described to be decreased in patients with MCI in different regions, including the bilateral medial frontal gyrus [19, 35, 60, 61]. This region is involved in executive function, attention, and memory [66], and therefore alterations in its connectivity might be associated with the AD-related cognitive impairments. Moreover, in the right nucleus accumbens, a significant decrease in FD-w strength was observed in transgenic rats compared with the control group, with a clear dependence on age. In fact, the age at MRI acquisition was directly related to the number of sessions required to achieve the criteria. Along this line, the nucleus accumbens plays an important role in motivation and reward processes [67], and therefore the lower connection strength of this region in the animals scanned at a later age could be related to their performance in the cognitive training. Indeed, higher connectivity in this area could be related to higher motivation and response to the positive stimuli (food) during training, leading to a shorter learning phase. Other studies have also related alterations in the reward system with AD. For instance, lower nodal efficiency in patients with AD and patients with MCI has been demonstrated in the caudate nucleus, a region also belonging to the reward system [60]. Similarly, a decrease in clustering coefficient in the subcallosal cortex with aging has been described previously, which is sharper in* APOE-ε4* carriers than in noncarriers [68], pointing to the relationship of alterations in this region with a higher risk for AD.

Studies in MCI or AD human cohorts have shown altered structural network properties in other regions [19, 21, 35, 64], pointing to global network reorganization associated with the disease. In our study, we observed that some of these connectivity alterations are already present at very early periods, when the Aβ plaque concentration and other pathological landmarks are not yet significant [8].

### Functional connectivity alterations

Connectomics studies in humans have shown differences between AD and control functional networks, although not always in the same direction. Thus, characteristic path length has been shown to be both decreased [69, 70] and increased [38, 71, 72] in patients with AD and patients with MCI. Likewise, a decrease in global efficiency (inversely related to characteristic path length) associated with AD has been described [73–75]. AD or risk for AD has been associated with both a decrease [31, 38, 76] and an increase [69, 70] in the clustering coefficient. The inconsistency observed between these studies could be explained by methodological issues related to the construction of the connectivity matrix (Pearson’s correlations, partial correlations, or synchronization likelihood being some of the different methodologies considered) and the characterization of the network links (with some studies using binary connectomes and others based on weighted connections) [77].

In our study, significant differences in the global functional network metrics were not found. Indeed, the influence of the connectome definition in the comparison between transgenic and control rats was patent, as shown in Fig. 5. Whereas the weighted connectome showed a (nonsignificant) trend toward decreased network metrics in AD, the opposite was observed in the binary connectome. Binary connectivity is related to the number of regions connected among them, whereas the weighted connectome takes into account the strength of the connections. Therefore, on one hand, these tendencies could be explained as the need for a higher number of connections to compensate for weaker correlations between the activity in different regions in the transgenic cohort. Further analysis with bigger cohorts should be performed to confirm these observations. On the other hand, the absence of significant differences could be related to two additional factors: first, the early stage of the disease that we were evaluating, and second, the effect of the training phase in the resting-state connectome. Learning-induced changes in resting-state connectivity have been described previously [78]. In our study, all the animals underwent cognitive training until they achieved a similar performance (acquisition criteria); therefore, this learning period could lead to similar functional network properties in both transgenic and control cohorts. This could be associated with a reorganization of functional connections to compensate for the differences in structure previously described.

Indeed, significant regional alterations were observed in the right insular cortex (decreased nodal strength), right amygdala (decreased binary nodal efficiency and clustering), and right hypothalamus medial tegmental area and VTA (increased nodal efficiency and clustering). Similarly to the structural connectome, areas related to the reward circuit, such as the amygdala and VTA [67], showed differences in network metrics between AD and control animals. These areas are also related to motivation and memory, which are altered in patients with AD, and have a major role in the neurocognitive tasks performed by the animals in our study. Specifically, in the case of AD, animals with lower values of the network parameters took longer to overcome the training phase, suggesting a direct relationship of the functional connectivity of these areas with learning abilities. Likewise, the lower connectivity found in the insular cortex of AD rats could also be related to the worse performance in training, because the insular cortex functions include perception, motor control, and cognitive functioning [67, 79], which also had a great impact in the DNMS training and testing the animals underwent. In line with our results, the amygdala and insula have previously been identified as regions affected by AD or MCI in functional connectomics [74, 80] and have been included between the most discriminant regions when developing classifiers for early AD [72, 81–83].

### Relationship between brain networks and cognitive function

From a whole-brain perspective, a strong correlation was observed between the learning abilities evaluated in the training phase and the local efficiency and strength of the FD-w network. Lower values of these metrics, involving less segregation and connection in the brain, were associated with more training sessions required to achieve the acquisition criteria. In the DNMS testing, the high correlation between global network metrics and cognitive performance disappeared, revealing an impact of these parameters in learning skills rather than in working memory abilities. The relationship between structural network metrics and cognitive performance has also been described in human populations. For instance, patients with AD performed worse than healthy subjects in verbal memory tests, and this performance was associated with lower local and global efficiency of the structural networks [21].

Regarding global functional network metrics, no correlations were found with training or testing results. As previously mentioned, learning has been shown to induce changes in resting-state networks [78]. Therefore, we hypothesize that the training period could have induced a global functional network organization that overcame deficits associated with the differences in structural networks. As a consequence, when the rats were scanned after the training and testing phases, the functional network organization in both groups was homogeneous at a global level, as were the percentage of correct responses in the DNMS phase, regardless of the number of training sessions required to achieve the learning criteria. Further studies are needed to elucidate these findings.

Nevertheless, when regional metrics were considered, both functional and structural properties of specific regions showed high correlations with the outcome of training or the DNMS protocol. In the same way that structural or functional alterations in connectivity were found in some areas of the reward circuit (nucleus accumbens and VTA), cognitive performance was highly correlated with some areas in this circuit, such as the septum and diagonal band, olfactory tubercle, and ventral pallidum. Higher-weighted structural or functional network metrics were correlated with faster achievement of criteria during training, suggesting that AD impairs connectivity in these areas, which are related to learning.

Regions involved in memory, learning, or perception functions such as the retrosplenial and entorhinal cortices, amygdala, or different areas of the hippocampus, were highly correlated with training performance. Several functional and structural network metrics were related to the number of training sessions required to achieve the criteria. The higher the metrics in these regions, the faster the learning of the task. These results are consistent with the fact that some of these regions are between the first areas affected by AD [8], and consequently memory and cognitive impairments would be linked to changes in their regional connectivity. Along this line, correlation between functional connectivity properties of the amygdala and cognition evaluated by Mini Mental State Examination scores were described previously [80]. Moreover, most of these regions belonging to the limbic system have important connections with prefrontal areas [84] whose structural connectivity was shown to be altered in the transgenic rats.

In addition, connectivity of brain areas with an important role in sensory function was also highly correlated with cognitive performance, namely the piriform cortex, olfactory nuclei and tubercle, and ventral hippocampus, all of them involved in olfactory functions. Of note, the structural and functional weighted metrics were anticorrelated with the number of sessions, whereas the binary functional metrics were positively correlated with both training sessions and the number of trials in the DNMS task. These results could be related to the different tendencies observed between functional weighted and binary metrics previously described. Together with olfactory-related regions, areas with a role in visual processing, such as temporal association and the visual cortex, were also correlated with cognition. Thus, higher structural network metrics of these two areas were associated with faster learning in the training phase.

### Strengths and limitations

This study provides characterization of the brain of a transgenic model of AD at an early period based on MRI-based connectomics. The use of MRI and graph theory to evaluate alterations associated with AD provides a highly translational approach because this methodology can easily be translated into clinical investigation. The TgF344-AD model shows all the pathological hallmarks of the disease in a progressive manner. This fact makes this model unique for the early characterization of the disease, which might contribute to the development of new biomarkers. The connectomics results of our study complement the previous histological characterization of brain changes [8, 55] and contribute to the understanding of AD as a disconnection syndrome. In addition, rat models allow for better behavioral characterization than other animal models [4]. Hence, a DNMS task was implemented in our study to characterize the learning and working memory skills starting at adolescence (2 months of age) and ending in early adulthood (5 months of age). This represents a very early stage in comparison with the previous behavioral studies performed in this model at 6 months of age [8, 55]. Our study has shown that, even at earlier periods, both cognitive and network properties are different between transgenic and control rats, and also that there is a correlation between the network metrics and cognitive performance.

Regarding the limitations of the study, we must highlight the small sample size that might hamper the statistical analysis. Nevertheless, significant differences were detected even with the small sample, which were coherent with those described in studies performed with bigger human cohorts.

However, because MRI sessions were performed just after the DNMS phase, there was certain variability in the age at which rats were scanned, which was related to the number of sessions required for training. To take this into account, age was included as a covariate in the analysis of network metrics. However, the dependence between network metrics and age could be related to two factors that are difficult to disentangle: first, brain maturation, and second, the fact that intrinsic connectivity properties impact performance in cognitive training, and therefore age at the time of scanning is also a consequence of differences in brain networks.

## Conclusions

AD has been described as a disconnection syndrome, and, in this study, connectivity disruption was identified in structural and functional networks of young adult TgF344-AD rats at as early a period as 5 months of age. At a global level, structural networks showed lower integration and segregation in transgenic than in control rats, pointing to a different pattern of anatomical connections in subjects developing AD. Structural connectivity differences did not lead to changes in global functional metrics, probably because of changes induced in the resting-state connectivity by the long cognitive training phase the animals underwent before MRI scanning. Differences in the functional or structural network properties were shown in several regions related to memory or reward circuit, known to be altered in patients with AD or MCI. Our results also indicate that connectivity of regions related to reward, memory, and sensory performance have an impact on the cognitive outcome of the animal. Regarding cognitive performance, impairments associated with the disease were observed, mainly related to the learning abilities at the training phase starting at 2 months of age. Therefore, this study suggests a pattern of alteration in the brain network with consequences in cognition already present at very early stages of the disease, when most of the pathological hallmarks have not yet been detected. The use of animal models for this early characterization represents a promising approach and points to the potential of MRI-based connectomics as an early biomarker of AD.

## Additional file

Additional file 1: Cognitive training description and evaluation. (DOC 135 kb)

## Abbreviations

AD: Alzheimer’s disease
ADHc: Anterodorsal hippocampus
ANT: Advanced normalization tool
Aβ: β-Amyloid
CC: Clustering coefficient
CSF: Cerebrospinal fluid
DB: Diagonal band
DNMS: Delayed nonmatch to sample
DWI: Diffusion-weighted imaging
Ent Cx: Entorhinal cortex
EPI: Spin echo planar imaging
FA: Fractional anisotropy
FA-w: Fractional anisotropy-weighted
FD: Fiber density
FDR: False discovery rate
FD-w: Fiber density-weighted
FOV: Field of view
GM: Gray matter
IPAC: Interstitial nucleus of the posterior limb of the anterior commissure
L: Left
LH: Lateral hypothalamus
MCI: Mild cognitive impairment
MDEFT: Modified driven equilibrium transform
MRI: Magnetic resonance imaging
NE: Nodal efficiency
Pir CX: Piriform cortex
R: Right
RARE: Rapid acquisition with relaxation enhancement
RS: Retrosplenial cortex
rs-fMRI: Resting-state functional magnetic resonance imaging
SI: Substantia innominata
TE: Echo time
TeA Cx: Temporal association cortex
TPM: Tissue probability map
TR: Repetition time
Tu: Olfactory tubercle
VCI: Vascular cognitive impairment
VCx: Visual cortex
VHc: Ventral hippocampus
VP: Ventral pallidum
VTA: Ventral tegmental area
WM: White matter
