# Supplementary material for: Early brain connectivity alterations and cognitive impairment in a rat model of Alzheimer’s disease
Source: Alzheimers Res Ther. 2018 Feb 7;10:16. doi: 10.1186/s13195-018-0346-2 (PMC5803915; doi:10.1186/s13195-018-0346-2)
Supplement: Additional file 1: — Cognitive training description and evaluation. (DOC 135 kb) [file 13195_2018_346_MOESM1_ESM.doc]

**Additional file**

***Cognitive function evaluation. Training and habituation.***

The training and DNMS (delayed non-matching-to-sample) task took place in operant chambers (Med Associates, USA) with a pellet dispenser and three retractile levers, two of them in the same chamber side where the feeder is (namely right and left levers) and the other in the opposite side of the chamber (center lever). During the behavioral testing weeks, rats were food-deprived, receiving only 75% of their usual food intake. Before DNMS, the animals underwent habituation and training stages.

*Habituation*

Before the protocol starts, all rats were handled 5 minutes daily for a week by the investigator responsible for all the DNMS procedure in order to decrease the rat stress levels. It was followed by a habituation stage where the animals stayed 30 minutes inside the operant chambers. After that a training is performed which consists of six stages with increasing difficulty.

*Training*

The first four stages are aimed to get the animal familiarized with the mechanism of pressing levers and get a pellet as reward: at first stage any lever is exposed and working; in the second stage the lever retract once pressed and then appears again; in the third stage the animal has to change between levers since they only work three times in a row; and in the fourth stage only the left or right lever (randomly) is exposed and the rat has to press 30 times each lever. Stages 1 and 2 had a fixed number of sessions, while 3 and 4 are repeated until a given number of pellets is obtained by the animal during the experiment. This criteria is a modification of the protocol in [1] in order to ensure that the rats learned the basics of the protocol before increasing difficulty in the next training steps.

Training stages 5 is similar to the DNMS protocol but with no delay between levers. And finally, training stage 6 introduced a random delay of 1 to 5 seconds between the levers. In these two phases the animals must achieve an acquisition criteria to start the next stage. In both cases, the criteria consisted on performing 2 consecutive days with a score of minimum 80% of correct responses. The number of sessions required to reach the criteria is recorded.

***Supplementary figures***

**Figure S1. Performance in the training stage 6 and relation between training and test performance.**


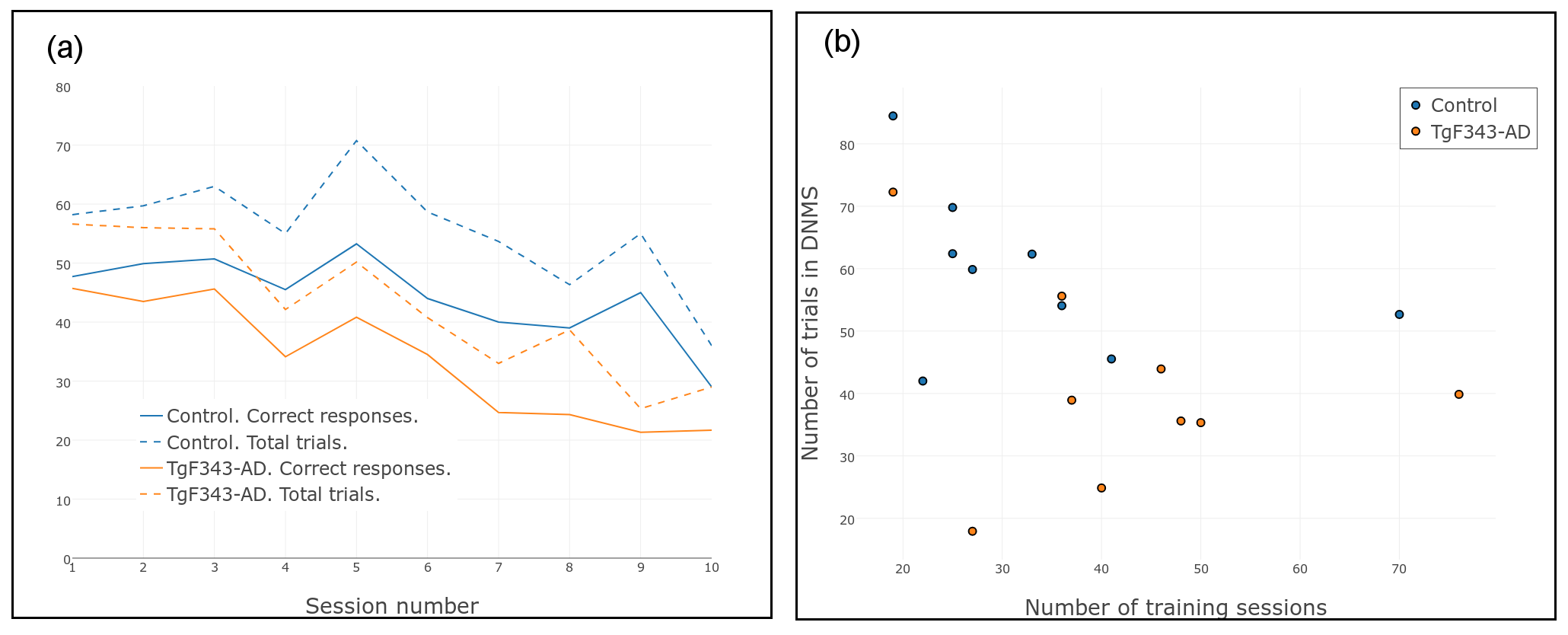


(a) Average number of trials (dashed line) and correct responses (solid line) in each session of the training phase 6 in the control (blue) and transgenic (orange) groups; (b) Relation between the number of training sessions required to achieve the criteria and the number of trials performed in the DNMS phase. Each dot corresponds to a subject (blue: control, orange: TgF343-AD)

1. Callaghan CK, Hok V, Della-Chiesa A, Virley DJ, Upton N, O’Mara SM. Age-related declines in delayed non-match-to-sample performance (DNMS) are reversed by the novel 5HT6 receptor antagonist SB742457. Neuropharmacology. 2012;63:890–7.
